# An inversion-based clustering approach for complex clusters

**DOI:** 10.1186/s13104-024-06791-y

**Published:** 2024-05-12

**Authors:** Mohammad Mahdi Barati Jozan, Aynaz Lotfata, Howard J. Hamilton, Hamed Tabesh

**Affiliations:** 1 Department of Medical Informatics, Faculty of Medicine, Mashhad University of Medical Sciences, Mashhad, Iran; 2 Department of Pathology, Microbiology, and Immunology, School Of Veterinary Medicine, University of California, Davis, USA; 3 Department of Computer Science, University of Regina, Regina, SK Canada

**Keywords:** Clustering algorithm, Inversion-based similarity measure, Overlapping clusters, Adjusted Rand index

## Abstract

**Background:**

The choice of an appropriate similarity measure plays a pivotal role in the effectiveness of clustering algorithms. However, many conventional measures rely solely on feature values to evaluate the similarity between objects to be clustered. Furthermore, the assumption of feature independence, while valid in certain scenarios, does not hold true for all real-world problems. Hence, considering alternative similarity measures that account for inter-dependencies among features can enhance the effectiveness of clustering in various applications.

**Methods:**

In this paper, we present the *Inv* measure, a novel similarity measure founded on the concept of inversion. The *Inv* measure considers the significance of features, the values of all object features, and the feature values of other objects, leading to a comprehensive and precise evaluation of similarity. To assess the performance of our proposed clustering approach that incorporates the *Inv* measure, we evaluate it on simulated data using the adjusted Rand index.

**Results:**

The simulation results strongly indicate that inversion-based clustering outperforms other methods in scenarios where clusters are complex, i.e., apparently highly overlapped. This showcases the practicality and effectiveness of the proposed approach, making it a valuable choice for applications that involve complex clusters across various domains.

**Conclusions:**

The inversion-based clustering approach may hold significant value in the healthcare industry, offering possible benefits in tasks like hospital ranking, treatment improvement, and high-risk patient identification. In social media analysis, it may prove valuable for trend detection, sentiment analysis, and user profiling. E-commerce may be able to utilize the approach for product recommendation and customer segmentation. The manufacturing sector may benefit from improved quality control, process optimization, and predictive maintenance. Additionally, the approach may be applied to traffic management and fleet optimization in the transportation domain. Its versatility and effectiveness make it a promising solution for diverse fields, providing valuable insights and optimization opportunities for complex and dynamic data analysis tasks.

**Supplementary Information:**

The online version contains supplementary material available at 10.1186/s13104-024-06791-y.

## Introduction

Clustering is a fundamental technique in data mining and machine learning, aiming to group objects into distinct clusters [1–7]. Objects within a cluster show high similarity to each other and low similarity to objects in other clusters, determined by a similarity measure [8–11].

Selecting an appropriate similarity measure is crucial for clustering algorithms. Studies indicate its significance in algorithm performance [8–12]. Various research assesses similarity measures across disciplines like web clustering [13], trajectory clustering [14], and chemical databases [15]. Some research endeavors have further examined performance variations based on the type of data being analyzed, differentiating between categorical data [16] and continuous data [8]. Consequently, some scholars [17] have advocated for the utilization of two fundamental clustering techniques: "similarity measures" for qualitative data and "distance measures" for quantitative data [17]. In this study, we'll refer to both types of measures as "similarity measures."

Table 1 lists similarity functions for qualitative data, and Table 2 shows distance functions for quantitative data.

**Table 1:** Some similarity functions for qualitative data [17]

Name	Function formula or measure method	Explanation
Jacard similarity	$J\left(A, B\right)= \frac{\left|A\cap B\right|}{\left|AUB\right|}$	1. Measures the similarity of two sets 2. $\left|X\right| \mathrm{is the number of elements of set} X$ 3. Jacard distance = 1 – Jacard similarity
Hamming similarity	The minimum number of substitutions needed to change one data point into the other	1. Smaller numbers indicate greater similarity 2. Hamming distance is the opposite of Hamming similarity
For data of mixed type	Map the feature into (0,1) Transform the feature into a dichotomous one ${S}_{ij}= \frac{1}{d} \sum_{l=1}^{d}{S}_{ijl}$ ${S}_{ij}= \left({\sum }_{l=1}^{d}{\gamma }_{ijl}{S}_{ijl}\right) / \left({\sum }_{l=1}^{d}{\gamma }_{ijl}\right)$

**Table 2:** Some distance functions for quantitative data [17]

Approach	Formula	Explanation
Minkowski distance	${\left(\sum_{l=1}^{d}{\left|{x}_{il}- {x}_{jl}\right|}^{n}\right)}^{1/n}$	A set of definitions for distance 1. City-block when n = 1 2. Euclidean distance when n = 2 3. Chebyshev distance when n $\to \infty$
Standardized Euclidean distance	${\left(\sum_{l=1}^{d}{\left|\frac{{x}_{il}- {x}_{jl}}{{s}_{l}}\right|}^{2}\right)}^{1/2}$	1. *s* stands for the standard deviation 2. A weighted Euclidean distance on the deviation
Cosine distance	$1-{\text{cos}}\alpha =\frac{Cov\left({x}_{i}.{x}_{j}\right)}{\sqrt{D({x}_{i})}\sqrt{D({x}_{j})}}$	1. *Cov* stands for the covariance and *D* stands for the variance 2. Measures the distance based on linear correlation
Pearson correlation distance	$1-\frac{Cov\left({x}_{i}.{x}_{j}\right)}{\sqrt{D({x}_{i})}\sqrt{D({x}_{j})}}$	1. *Cov* stands for the covariance and *D* stands for the variance 2. Measures the distance based on linear correlation
Mahalanobis distance	$\sqrt{{({x}_{i}- {x}_{j})}^{T}{S}^{-1}{({x}_{i}- {x}_{j})}}$	1. *S* is the covariance matrix inside the cluster 2. Has high computational complexity

A key limitation of similarity measures (e.g., Euclidean distance [17] and Hamming similarity [18]) is their exclusive reliance on feature values. Consequently, when two objects or entities exhibit similar feature values, they are considered more similar, regardless of any other pertinent factors. This oversimplification may overlook crucial aspects of the data.

Secondly, similarity measures often assume feature independence, neglecting their interdependence and potential influence on each other's values. This oversight may result in incomplete representations of data relationships. Moreover, most measures overlook feature prioritization, disregarding the varying importance of features in determining similarity. These assumptions do not fully align with real-world complexities, potentially limiting applicability and accuracy. To address these challenges, researchers explore inversion as a promising approach, investigating its theoretical and practical aspects [19–22].

In one study [19], a new constructive bijection connects permutations with a specific number of inversions to those with a particular major index, facilitating exploration of mathematical connections. Another work [20] introduces a probability distribution on the group of permutations of the set of integers, providing insights into inversion's probabilistic aspects for permutation-based data analysis. Furthermore, [21] presents six bijections linking a specific type of polyominoes called deco polyominoes with permutations, establishing connections between classical statistics and permutation-related analyses. Moreover, [22] proposes an efficient solution for counting interior edge crossings in bipartite graphs, relevant for layered graph drawing and data visualization enhancement.

This study introduces "*Inv*," an inversion-based similarity measure addressing previous challenges. It forms the basis of a new clustering approach grouping objects by inversion-based similarity. The primary goal is to optimize clustering by maximizing intra-cluster similarity and minimizing inter-cluster similarities. By incorporating this measure, we anticipate achieving more meaningful clustering results that better reflect underlying data patterns and relationships.

## Data and method

**Fig. 1:**
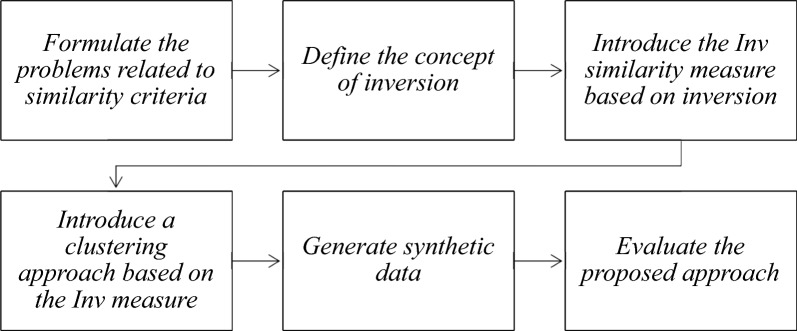
Flowchart of implementation and evaluation of the inversion-based clustering approach

The flowchart of the activities undertaken to assess the new inversion-based clustering approach is presented in the Fig. 1.

### Formulation of challenges related to similarity criteria

Several key terms used in simulation examples are defined below.

*Object:* Any entity that we intend to cluster is an *object*. Each object ${obj}_{i}$ has *n* features and can be represented by a vector: ${obj}_{i}= \langle {f}_{i1}, {f}_{i2}, \dots , {f}_{in}\rangle$.

*Feature space:* Each feature has a valid range of values known as its *feature space*, represented by ${S}_{f}$ for feature $f$.

*Universe:* The set of all objects we want to cluster is known as the *universe*, represented by $U= \left\{{obj}_{1}, {obj}_{2},\dots ,{obj}_{k}\right\}.$

*Similarity Measure:* A *similarity measure* is a measure that takes two objects as input and outputs a numeric value representing their similarity.

*Clustering problem:* A problem of partitioning *k* objects into *m* groups $\langle {C}_{1}, {C}_{2}, \dots , {C}_{m}\rangle$ according to a specified similarity measure, so that the objects in each group are as similar as possible to each other and as different as possible from objects in other groups. These clusters adhere to two constraints [1–7]:

1 $$\bigcup_{i=1}^{m}{C}_{i}=U$$

2 $${C}_{i}\bigcap_{\begin{array}{c}j=1..m\\ i\ne j\end{array}}^{i=1..m}{C}_{j}= \varnothing$$

In the following paragraphs, the challenges stated in the introduction section are formulated using the defined terms.

#### Symmetry challenge

A drawback of distance-based measures is called the *Symmetry challenge*, where adding or subtracting a value to a feature has the same distance effect. Given that Euclidean distance is widely used in clustering algorithms [23], it is used as a representative of distance-based measures in the following practices. Practice 1 demonstrates the weakness of similarity criteria when adding or subtracting a specific value to a feature.

*Practice 1:* Consider the following three objects:

$${obj}_{1}=\langle {V}_{1}, {V}_{2}, \dots , {V}_{n}\rangle$$

$${obj}_{2}= \langle {V}_{1}+v, {V}_{2}, \dots , {V}_{n}\rangle$$

$${obj}_{3}= \langle {V}_{1}-v ,{V}_{2}, \dots , {V}_{n}\rangle$$

where

3 $${V}_{1},{V}_{1}+v, {V}_{1}-v \in {S}_{{f}_{1}}$$

The first feature of ${obj}_{2}$ is *v* units less that the first feature of ${obj}_{1}$, and the first feature of ${obj}_{3}$ is *v* units more than the first feature of ${obj}_{1}$. All other features are equal in the three objects. The Euclidean distance $E$ between them, the similarity for adding $v$ to the first feature is calculated as follows:

4 $$\begin{aligned}E\left({Obj}_{1},{ Obj}_{2}\right)=&\sqrt[2]{{\left({V}_{1}- {(V}_{1}+v)\right)}^{2}+{\left({V}_{2}- {V}_{2}\right)}^{2}+\dots + {\left({V}_{n}- {V}_{n}\right)}^{2} }\\=& \sqrt[2]{{\left({V}_{1}- {V}_{1}-v\right)}^{2}+{\left({V}_{2}- {V}_{2}\right)}^{2}+\dots + {\left({V}_{n}- {V}_{n}\right)}^{2} }=v\end{aligned}$$

and the similarity for subtracting $v$ from the first feature is calculated as follows:

5 $$\begin{aligned}E\left({Obj}_{1},{ Obj}_{3}\right)=&\sqrt[2]{{\left({V}_{1}- {(V}_{1}-v)\right)}^{2}+{\left({V}_{2}- {V}_{2}\right)}^{2}+\dots + {\left({V}_{n}- {V}_{n}\right)}^{2} }\\=&\sqrt[2]{{\left({V}_{1}- {V}_{1}+v\right)}^{2}+{\left({V}_{2}- {V}_{2}\right)}^{2}+\dots + {\left({V}_{n}- {V}_{n}\right)}^{2} }=v\end{aligned}$$

This challenge can arise for any feature. In general, this challenge can be expressed as follows:

$${obj}_{1}= \langle {V}_{1}, {V}_{2}, {\dots , {V}_{i},\dots ,V}_{n}\rangle$$

$${obj}_{2}= \langle {V}_{1}, {V}_{2},\dots , {V}_{i}+v,\dots , {V}_{n}\rangle$$

$${obj}_{3}= \langle {V}_{1},{V}_{2}, \dots , {V}_{i}-v,\dots , {V}_{n}\rangle$$

$$E \left({obj}_{1} , {obj}_{2}\right)= E\left({obj}_{1}, {obj}_{3}\right)$$

where

6 $$\begin{aligned}&{V}_{i},{V}_{i}+v,{V}_{i}-v\in {S}_{{f}_{i}}\\&1\le i\le n\end{aligned}$$

The equality of the distances is mathematically correct, but in the real world, the significance of adding a specific value to a feature may be different from that of subtracting the same amount. An example based on student scores can be found as Supplementary Example 1 (see Additional file 1).

#### Place symmetry challenge

The *Place Symmetry challenge* is a generalized form of the previous challenge. In this challenge, increasing or decreasing a value can occur for each feature. In Practice 2, the weakness of the similarity criteria when adding or subtracting a certain value to possibly different features of objects is shown.

*Practice 2:* Consider the following three objects:

$${obj}_{1}= \langle {V}_{1}, {V}_{2}, \dots , {V}_{n}\rangle$$

$${obj}_{2}= \langle {V}_{1}, {V}_{2},\dots , {V}_{i}+v ,\dots , {V}_{n}\rangle$$

$${obj}_{3}= \langle {V}_{1},{V}_{2}, \dots , {V}_{j}-v,\dots , {V}_{n}\rangle$$

$$E \left({obj}_{1} , {obj}_{2}\right)= E\left({obj}_{1}, {obj}_{3}\right)$$

where

7 $$\begin{aligned}&{V}_{i},{V}_{i}+v,{V}_{i}-v\in {S}_{{f}_{i}}\\&{V}_{j},{V}_{j}-v\in {S}_{{f}_{j}}\\&1\le i,j\le n\end{aligned}$$

if ${V}_{i}$ represents age and ${V}_{j}$ represents weight, both normalized to their respective feature spaces, adding $v$ units to ${V}_{i}$ and subtracting $v$ units from ${V}_{j}$ yields the same Euclidean distance between two pairs of objects. However, the implications of increasing or decreasing $v$ units in weight differ from those in age due to varying value distributions. Hence, altering their values by $v$ holds distinct meanings for each feature.

#### Feature independence challenge

The Feature Independence challenge refers to the treatment of features as if they are independent of each other when calculating distance-based measures, whereas in practice they are often interdependent.

### Definition of the concept of inversion

To address the challenges defined in section "Formulation of challenges related to similarity criteria", a similarity measure named *Inv*, which is based on the concept of inversion, is proposed. The mathematical definition of inversion is as follows:

*Inversion:* In a sequence $S= \langle {a}_{1}, {a}_{2}, \dots , {a}_{n}\rangle$ of pairwise comparable elements ${a}_{i} (i=\mathrm{1,2},\dots ,n)$, a pair $({a}_{i},{a}_{j})$ is called an *inversion* if $i<j$ and ${a}_{i}>{a}_{j}$ [22].

The concept of inversion is illustrated by Practice 3.

*Practice 3:* Suppose we have five movies and we ask two people to rate them on a scale of 1 to 10 according to their preferences. In this example, an object is a *person* and it is represented by a vector with 5 features [24].

8 $$person= \langle { Score(Movie}_{1}),{ Score(Movie}_{2}), { Score(Movie}_{3}), { Score(Movie}_{4}), { Score(Movie}_{5})\rangle$$

Scores are given in Table 3**.**

**Table 3:** Users’ ratings of movies

Movies	Score of person 1	Score of person 2
Movie1	10	9
Movie2	6	3
Movie3	5	9
Movie4	1	6
Movie5	8	1

The definition of inversion for only one person is presented in Supplementary Example 1 (see Additional file 1). Let's now explore a more indirect application of the definition to determine the inversions between the scores of the two people. We arrange the movies for each person in a rank sequence based on preferences, with the highest-scoring movie first. Ties are resolved by ranking the movie with the lower movie number first. Person 1's sequence is $\langle {Movie}_{1}, {Movie}_{5},{Movie}_{2},{Movie}_{3},{Movie}_{4}\rangle$, while Person 2's is $\langle {Movie}_{1}, {Movie}_{3},{Movie}_{4},{Movie}_{2},{Movie}_{5}\rangle$. Renaming the movies based on Person 1's rank sequence, we get 〈1,2,3,4,5〉 for Person 1 and 〈1,4,5,3,2〉 for Person 2. Applying the inversion definition to the second sequence, we find 4 is inverted compared to 3 and 2 (two inversions), 5 is inverted compared to 3 and 2 (two inversions), and 3 is inverted compared to 2 (one inversion), totaling five inversions.

A visual method for counting inversions involves organizing movies for each person by their scores, highest to lowest, and connecting corresponding movies. The intersections of these lines indicate the number of inversions between the two sequences.

**Fig. 2:**
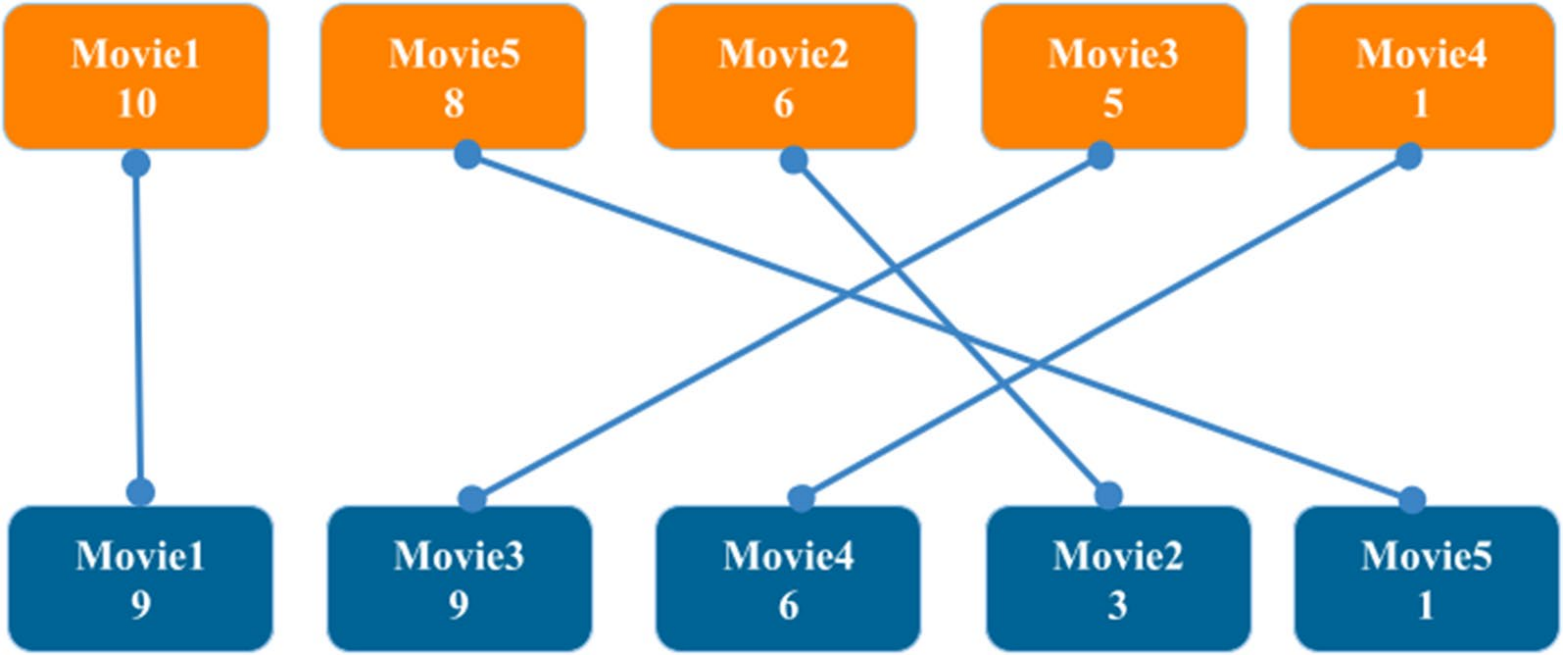
Each point of intersection between lines representing an inversion (practice 3)

In Fig. 2**,** the five inversions are indicated by the five points where the lines intersect. The more different the sequences, the greater the number of the inversions. The minimum and maximum number of inversions of two vectors with *n* features are 0 and $\frac{n\left(n+1\right)}{2}$, respectively [25]. Supplementary Code 1 contains the inversion calculation algorithm (see Additional file 1).

Notice from *Practice3* that the way ties are broken affects the number of inversions. The proposed way of resolving ties is to consider a default sequence for features, and when the values of two or more features are the same, their order will be based on the default order. For instance, in the movies database, priority is given to movies with smaller numbers. Thus, as Person 2 rated Movie 1 and Movie 3 equally at 9, Movie 1 takes precedence in the rank order.

*Practice3* illustrates the importance of feature prioritization in cases where values are equal. Depending on specific requirements, certain features may need to be prioritized over others, which can have a considerable impact on the resulting number of inversions.

### Introduction of the *Inv* similarity measure based on the notion of inversion

In this subsection, the *Inv* inversion-based similarity measure is introduced. The *Inv* measure of the similarity of two objects is defined as the number of inversions that exist between two objects according to the *Count_Inversions* function. Formally, the measure is defined as follows:

$${obj}_{1}= \langle {V}_{11}, {V}_{12}, \dots , {V}_{1n}\rangle$$

$${obj}_{2}= \langle {V}_{21}, {V}_{22},\dots , {V}_{2n}\rangle$$

9 $$Inv({obj}_{1},{obj}_{2}) =Count\_Inversions\left({obj}_{1},{obj}_{2}\right)$$

According to the *Inv* measure, as inversion count between two sequences rises, their similarity decreases, and vice versa. Although we refer to inversion as a similarity measure, it actually yields a measure of dissimilarity because the greater the similarity between two objects, the lower the number of inversions.

Three advantages of the inversion-based similarity measure are as follows [22]: the number of inversions is affected by the rank positions of the features in the vector (Feature Independence challenge); the number of inversions when *v* is added to feature ${f}_{i}$ can be different from the number of inversions when it is subtracted from ${f}_{i}$ (Symmetry challenge); and the number of inversions when *v* is added to ${f}_{i}$ may not be the same as the number of inversions when *v* is added to ${f}_{j}$ (Place Symmetry challenge).

The Symmetry challenge in distance-based measures results from the equal impact of adding or subtracting a value from a feature on the distance. In calculating inversions, not only is the value of a particular feature considered, but also the values of other features become integral. The feature's rank position within the object's vector significantly affects the inversion count. Altering a feature's rank position by adding a value may yield different outcomes compared to subtracting the same value, influenced by other features. Consequently, the inversion count varies depending on specific features, emphasizing the nuanced nature of inversion calculations.

The Place Symmetry challenge expands upon the Symmetry challenge by allowing the increase or decrease of a value for each feature individually. As previously noted, the count of inversions is influenced by both the value of a specific feature and other features, so it addresses this challenge as well as the previous challenge.

To challenge the independence of features, since determining the number of inversions requires determining the values and order of all features, hence the features cannot be independent of each other. The explanation of how the proposed measure addresses the outlined challenges are illustrated in Supplementary practice 1 (see Additional file 1).

The proposed similarity measure offers an additional advantage by allowing adjustments to consider feature importance in distance calculation. This enables the consideration of feature relevance or priority, enhancing the measure's utility. To accommodate priorities, the inversion measure can be modified to incorporate both feature value and assigned priority. By default, all variables are assumed to have equal priority. *Practice 4* demonstrates a method for factoring feature priority into similarity calculation.

**Fig. 3:**
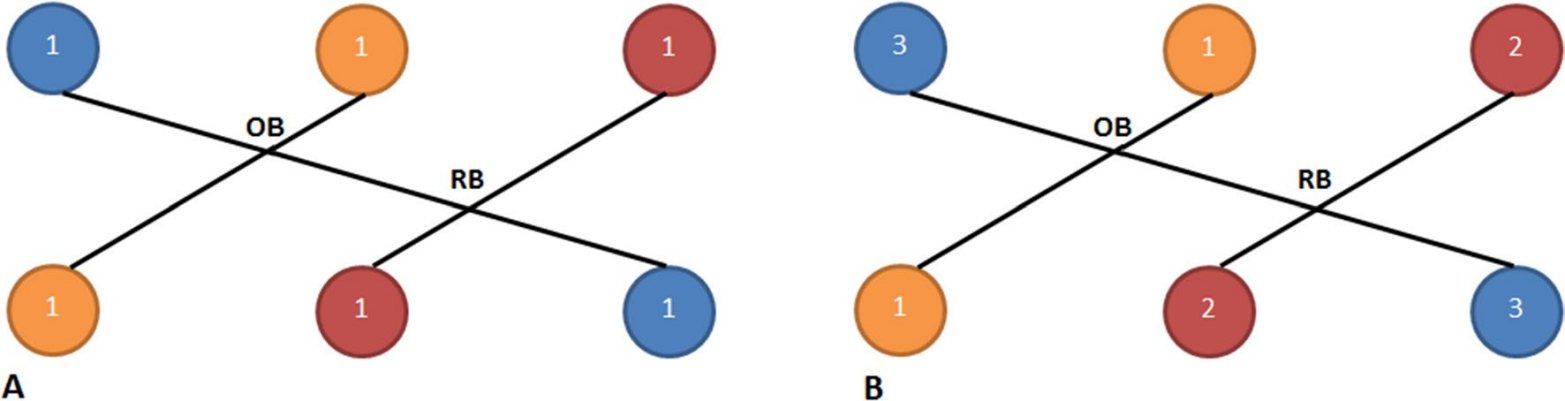
Prioritizing features: **A** All features have the same priority, which is one, **B** The priority of each feature is displayed within the corresponding circle (Practice 4)

*Practice 4***:** Consider two objects, each with three features, depicted by colored circles in Fig. 3. Let the orange feature have priority 1 (lowest), the red feature priority 2, and the blue feature priority 3 (highest). An adjusted inversion function is employed, where each inversion detected is weighted by the product of the relevant features' priorities.

According to Fig. 3A, there are 2 inversions because there are 2 points where the lines intersect (OB and RB). Since the priority of every feature is one, the adjusted inversion function is as calculated as:

10 $$\begin{aligned}AdjInv({\text{Figure 3}}\_\text{A})=&OB+RB\\=&1\left(1\right)+1\left(1\right)=2\end{aligned}$$

To calculate the adjusted inversion for Fig. 3B, the products of the priorities of each inversion (represented by a line) are also considered.

11 $$\begin{aligned}AdjInv({\text{Figure 3}}\_\text{b})=&OB+RB\\&=1\left(3\right)+2\left(3\right)=9\end{aligned}$$

### Introduction of the clustering approach based on the *Inv* measure

In this section, we formulate an algorithmic framework for an inversion-based clustering approach as a type of partitioning clustering method and then we instantiate the framework with different measures to specify two inversion-based clustering algorithms. The main steps of a partitioning clustering method are as follows. First, the number of desired clusters is chosen and initial centroids in the range of the feature values are randomly selected. Next, every input object is assigned to the nearest centroid based on its distance from it using the similarity measure. Every centroid is then moved to the mean location of its assigned cluster, and this process is iteratively repeated until convergence is reached, as indicated by no further changes in assignments [25]. The pseudo-code of proposed algorithmic framework can be found as Supplementary Code 2 (see Additional file 1).

#### Algorithmic framework for inversion-based clustering

The steps of the algorithmic framework are described below.

*Input:* $U=\{{obj}_{1}, {obj}_{2}, \dots , {obj}_{k}\}$, number of clusters ($m$)

*Output*: $$m clusters \left({C}_{1}, {C}_{2}, \dots , {C}_{m}\right)$$

*Step 1)* Normalize each feature based on the Min–Max Feature scaling method. The normalized feature value ${F}_{ia}$ for feature ${f}_{a}$ in object ${obj}_{i}$ is calculated as the following equation:

12 $${F}_{ia}=\frac{{f}_{ia}-{\text{min}}({f}_{a})}{{\text{max}}\left({f}_{a}\right)-{\text{min}}({f}_{a})}$$

where ${f}_{ia}$ is the original feature value for ${obj}_{i}$, ${\text{min}}({f}_{a})$ is the minimum value and ${\text{max}}\left({f}_{a}\right)$ is the maximum value of the feature across all objects in universe *U*, and ${F}_{ia}$ is the normalized value. The feature space for every normalized feature is equal to [0, 1].

*Step 2)* Initialize the centroids ${c}_{1}, {c}_{2}, \dots , {c}_{m}$ of clusters ${C}_{1}, {C}_{2}, \dots , {C}_{m}$ randomly.

*Step 3)* Calculate the number of inversions between every combination of an object ${obj}_{i}$ and a centroid ${c}_{j}$ and assign every object to the cluster for the centroid with the fewest inversions.

$$Assign \,{obj}_{i} \,to\, Cluster \,{C}_{b}, \,where \,b={\text{arg}}{min}_{j} \left(Measure\left({obj}_{i},{c}_{j}\right)\right)$$

*where*

13 $$1\le i\le k , 1\le j\le m$$

*Step 4)* For each cluster ${C}_{j}$, calculate the new centroid ${c}_{j}$ as the mean of the objects assigned to the cluster:

$${c}_{j}=\frac{1}{{N}_{j}}\sum_{c\in {C}_{j}}c$$

where

14 $$1\le j\le m$$

${N}_{j}$ is the number of objects assigned to cluster ${C}_{j}$, and the sum is taken over all objects in this cluster.

*Step 5)* Repeat Steps 3 and 4 until the clusters do not change, or the number of iterations reaches a predetermined value.

For the third step of the algorithmic framework, which is to assign an object to a cluster using a measure, two measures are proposed, which lead to the Inversion-Based Clustering Algorithm (ICA) and the Regulator Inversion Euclidean distance based Clustering Algorithm (RIECA).

#### Approach 1) Inversion-Based Clustering Algorithm (ICA)

In the first round of ICA, the centroids are randomly initialized, then inversions between every combination of an object and a centroid are calculated, and finally every object is assigned to the centroid with the fewest inversions. In subsequent rounds, inversions between every combination of an object and an updated centroid are calculated. In other words, the measure function is defined as follows:

15 $$Measure\left({obj}_{i},{c}_{c}\right)=Inv\left({obj}_{i},{c}_{c}\right)$$

where ${obj}_{i}$ is the ${i}^{th}$ object, ${C}_{c}$ is ${c}^{th}$ cluster (which has centroid ${c}_{c}$), and $Inv\left({obj}_{i},{c}_{c}\right)$ is the number of inversions between ${obj}_{i}$ and ${c}_{c}$.

#### Approach 2) Regulator Inversion Euclidean distance based Clustering Algorithm (RIECA)

With RIECA, inversion is used as a regulator for Euclidean distance [17], i.e. the Euclidean distance is multiplied by the number of inversions. If the number of inversions is high, the value of the measure grows more than if the number of inversions is low. In this approach, the *Measure* function is defined as follows:

$$Measure\left({Obj}_{i},{c}_{c}\right)=Inv\left({Obj}_{i},{c}_{c}\right)* E\left({Obj}_{i},{c}_{c}\right)$$

*where*

16 $$1\le i\le k , 1\le c\le m$$

where ${Obj}_{i}$ is the ${i}^{th}$ object, ${c}_{c}$ is the ${c}^{th}$ cluster, $Inv\left({Obj}_{i},{c}_{c}\right)$ is the number of inversions between ${Obj}_{i}$ and ${c}_{c}$, and $E\left({Obj}_{i},{c}_{c}\right)$ is the Euclidean distance between ${Obj}_{i}$ and ${c}_{c}$.

## Results

This section describes the generation of synthetic data and the evaluation of the proposed approach, which correspond to the final two steps of the flowchart given in section "Data and method".

### Generation of synthetic data

Synthetic datasets were generated using the MixSim package [26]. The average pairwise overlap parameter (denoted as ῶ) in package allows for the creation of clusters with varying degrees of complexity, ranging from well-separated (ῶ = 0.001) to highly overlapped ones (ῶ = 0.4) [27]. The summary of the package documentation can be found as Supplementary Documentation 1 (see Additional file 1).

To evaluate the performance of the ICA and REICA algorithms, we generated two types of random data samples for each value of ῶ (0.4, 0.3, 0.2, 0.05, and 0.001). The first type of data sample (referred to as Simulated Dataset1) comprised 200 random data values with 6 features, forming 5 clusters. The second type of data sample (referred to as Simulated Dataset2) included 500 random data values with 7 features, forming 10 clusters. These datasets facilitated comprehensive evaluation of algorithm performance across diverse clustering complexities.

### Evaluation of the proposed approach

The proposed ICA and REICA algorithms are compared with EM clustering [28] from the *mclust* package [29], k-means [3] and hierarchal clustering [30] from the *stats* package, and k-medoids [31] clustering from the *cluster* package [32]. For the comparison, 1000 data samples of Simulated Dataset 1 and 1000 of Simulated Dataset 2 were prepared and then each algorithm was run on each sample separately. The ICA and REICA algorithms are implemented in R version 3.4.2 [33]. This study employs the *adjusted Rand index* implemented in the MixSim package to evaluated algorithms [26, 34–36]. The values obtained for the adjusted Rand index for the algorithms when applied to Simulated Dataset 1 are shown in Table 4.

**Table 4:** Effectiveness of the algorithms in clustering 6-dimensional datasets of size 200 with 5 clusters (Simulated Dataset 1)

	0.4	0.3	0.2	0.05	0.001
EM clustering	$0.055\pm 0.027$	$0.097\pm$ 0.044	$0.176\pm 0.064$	$0.572\pm 0.098$	$0.981\pm 0.032$
k-means clustering	$0.066\pm$ 0.029	$0.119\pm 0.043$	$0.211\pm 0.061$	$\mathbf{0.622}\pm \mathbf{0.087}$	$\mathbf{0.983}\pm \mathbf{0.025}$
k-medoids clustering	$0.061\pm$ 0.028	$0.107\pm 0.039$	$0.187\pm 0.060$	$0.567\pm 0.092$	$0.975\pm 0.023$
Hierarchal clustering	$0.058\pm$ 0.027	$0.103\pm 0.40$	$0.185\pm 0.060$	$0.574\pm 0.091$	$0.979\pm 0.021$
ICA	$0.106\pm 0.029$	$0.156\pm 0.040$	$0.229\pm 0.054$	$0.494\pm 0.092$	$0.824\pm 0.093$
REICA	$\mathbf{0.109}\pm \mathbf{0.030}$	$\mathbf{0.167}\pm \mathbf{0.043}$	$\mathbf{0.253}\pm \mathbf{0.059}$	$0.584\pm 0.087$	$0.935\pm 0.046$

The most effective algorithm is highlighted in bold

Table 4 shows that for complex cluster structures (ῶ = 0.4, 0.3, and 0.2), REICA algorithm outperforms other algorithms in cluster formation. However, for moderately or highly separated clusters (ῶ = 0.05, 0.001), both ICA and REICA algorithms perform poorly compared to other algorithms. This was anticipated, as distance between objects outweighs the influence of inversions in such scenarios.

To evaluate the statistical significance of the adjusted Rand index differences among algorithms, we conducted a one-way ANOVA test. Further details are available in Supplementary statistical test result 1 (see Additional file 1).

Tables 5 and 6 reveal the optimal algorithm for each ῶ value, offering insights into algorithm performance across diverse clustering scenarios. These results elucidate the efficacy of ICA and REICA algorithms amidst varying clustering complexities and separations.

**Table 5:** The most effective algorithm in clustering 6-dimensional datasets of size 200 with 5 clusters (Simulated Dataset 1)

ῶ	The most effective algorithm
ῶ = 0.4	REICA, ICA
ῶ = 0.3	REICA
ῶ = 0.2	REICA
ῶ = 0.05	k-means
ῶ = 0.001	k-means, EM, k-mediods, hierarchical

**Table 6:** The most effective algorithm in clustering 7-dimensional datasets of size 500 with 10 clusters (Simulated Dataset 2)

ῶ	The most effective algorithm
ῶ = 0.4	REICA
ῶ = 0.3	REICA
ῶ = 0.2	REICA
ῶ = 0.05	k-means
ῶ = 0.001	${\text{EM}}$

In highly or moderately separated clusters, distance becomes more influential, diminishing the role of the number of inversions. Consequently, the proposed algorithms are less effective in handling such scenarios compared to other types of clustering. REICA excelled in complex structures, while no algorithm consistently outperformed others in highly or moderately separated clusters. When ῶ = 0.05, k-means was most effective in both simulations. Conversely, with ῶ = 0.001 and a small number of clusters and samples (Simulated Dataset 1), k-means, EM, K-medoids, and Hierarchical algorithms were highly effective. However, with a large number of clusters and samples (Simulated Dataset 2), EM algorithm proved most effective. These findings highlight algorithm strengths in diverse clustering conditions, revealing performance across varying complexities and separations. The selection of the optimal algorithm depends on specific data characteristics and nature.

## Discussion

Clustering techniques encompass partitioning, hierarchical, density-based, grid-based, and model-based methods [25]. The proposed algorithms fall under partitioning clustering. ICA is similar to k-means but utilizes an inversion-based similarity measure instead of a distance measure, whereas REICA utilizes inversion as a form of regulation for the Euclidean distance. In REICA, Euclidean distance is multiplied by the number of inversions. As a result, when there are more inversions, the measure value increases more prominently compared to scenarios with fewer inversions. This innovative method enriches the algorithm's ability for intricate cluster handling and enhances data analysis insights.

One strength of this study is that it identified three major challenges for Euclidean distance measures: the Symmetry challenge, the Place Symmetry challenge, and the Feature Independence Challenge. To address these challenges, we introduce the inversion-based measure "*Inv.*" Our findings show the significance of both Euclidean distance and inversions for similarity measures. Particularly, REICA, which multiplies inversions by Euclidean distance, outperforms ICA, which relies solely on inversions. The effectiveness of REICA, which employs a hybrid measure that considers inversions and Euclidean distance, suggests potential for developing other hybrid measures considering both factors. Such measures could prioritize inversion for complex clusters and emphasize Euclidean distance for well-separated clusters, aligning with k-means and other clustering methods.

Another strength of this study is that it benefits from applying statistical testing techniques like one-way ANOVA to rigorously assess the effectiveness of the proposed algorithms in comparison to existing algorithms. Additionally, simulating diverse data samples covering various clustering complexities enhances the findings' robustness and generalizability.

One strength of the proposed inversion-based algorithm is its ability to prioritize features differently. This prioritization operates at two levels. Firstly, during inversion calculation, equal feature values are sorted based on predetermined priority, influencing the number of inversions. Secondly, priority can be introduced as a coefficient in inversion calculations, where each intersection reflects the multiplication of feature priority values. As a result, the distance between two objects will increase with higher priority values, allowing more flexible and nuanced clustering.

For evaluation, the proposed algorithms' performance was compared with classical clustering methods: k-means [3], EM clustering [28], hierarchical clustering [30], and k-medoids [31]. Results indicate RIECA outperforms other algorithms with complex cluster structures [37, 38]. However, in scenarios of moderate or high cluster separation, the proposed algorithms are less effective, consistent with Berikov [39] and Cupertino [40].

A limitation of the inversion-based clustering approach is its effectiveness sensitivity to initial centroid selection, a common challenge in partition-based algorithms [25]. Mitigation strategies, such as repetition and employing the K-means++ initialization method [41], can be adapted to address this issue. In this study, we adopt the solution of running the algorithm several times with random initial centroids [25].

Another limitation is the lack of evaluation on real datasets. Assessing an algorithm's performance on actual data is challenging due to unknown cluster complexity. To address this, one could compare synthetic datasets with known complexity to real databases to gain an understanding of the complexity, then evaluate the algorithms under conditions similar to practical tasks. This approach offers insights into real-world performance, establishing their relevance and reliability for diverse data analysis scenarios.

## Conclusion

This paper emphasizes the significant impact of similarity measures on clustering algorithm performance. Existing measures, often reliant on feature values and assuming feature independence, may not yield optimal results in practice. To address this, we introduced the innovative inversion-based *Inv* measure, which considers other object and feature values through inversion. We proposed two algorithms (ICA and RIECA) based on *Inv* measure, and evaluated their performance using simulated data. Results showed inversion-based clustering outperformed traditional techniques for complex cluster structures.

Future studies can explore practical applications of the *Inv* measure in real-world problems to improve clustering performance across domains. Further research could investigate other hybrid measures combining inversions and distance-based measure.

### Supplementary Information

Additional file 1. Supplementary Figures, Tables and Codes. This file contains supplementary figures, tables and codes that provide further insights into the experimental setup and results discussed in the article.

## Data Availability

The datasets used and/or analyzed during the current study are available from the first author on reasonable request.
