# Supplementary material for: An inversion-based clustering approach for complex clusters
Source: BMC Res Notes. 2024 May 12;17:133. doi: 10.1186/s13104-024-06791-y (PMC11089746; doi:10.1186/s13104-024-06791-y)
Supplement: Supplementary file 1 — Additional file 1. Supplementary Figures, Tables and Codes. This file contains supplementary figures, tables and codes that provide further insights into the experimental setup and results discussed in the article. [file 13104_2024_6791_MOESM1_ESM.docx]

**Supplementary Example 1**

**Symmetry challenge:** Suppose the scores obtained by students in a Data Mining course give the box plot shown in Figure S1:


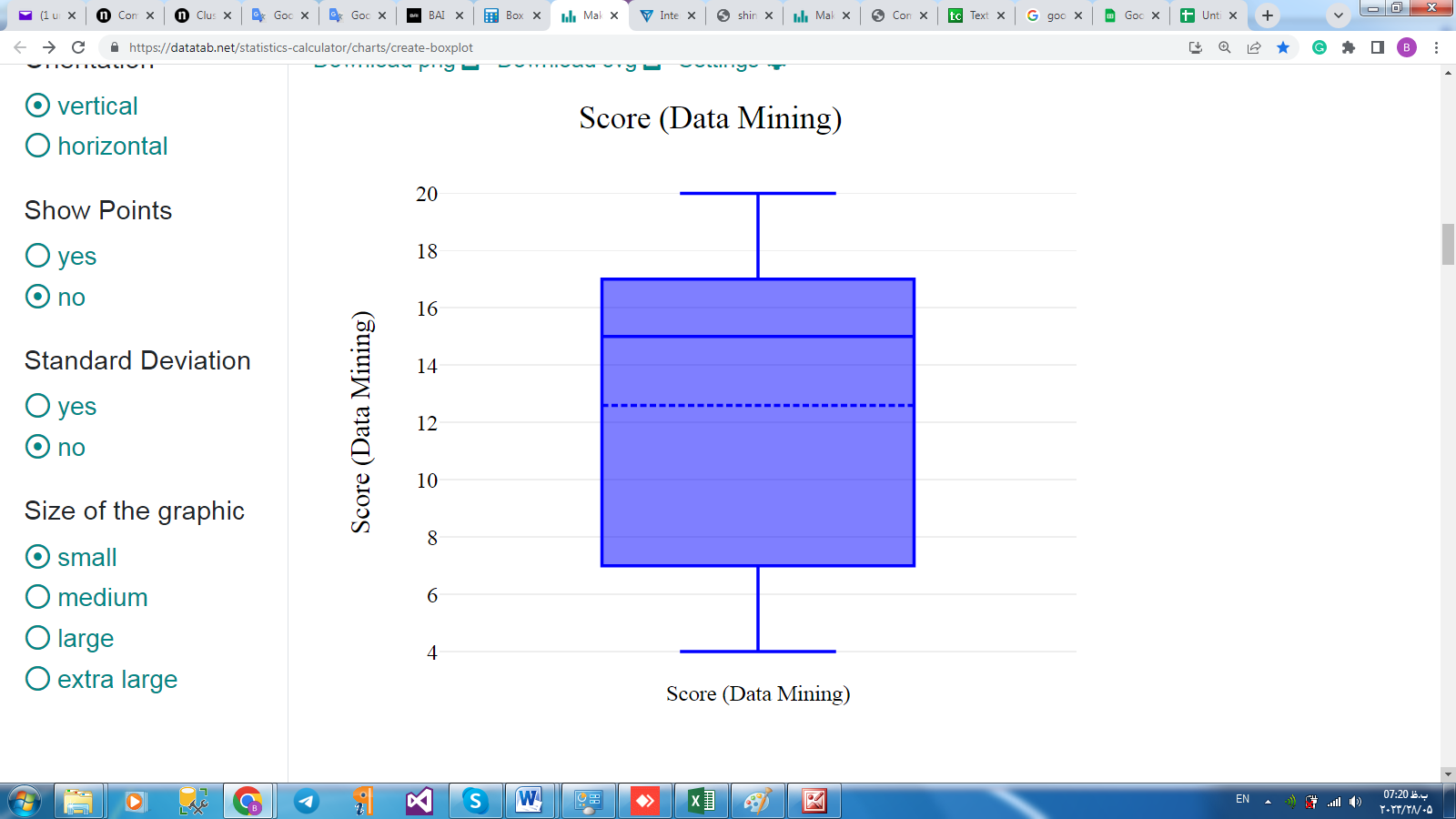


Figure S1 : The boxplot of the Data Mining course scores

Suppose the success of students is evaluated based on the quartile of their scores. If $V_{1}$ is equal to 14 and $v$ is equal to 2, then $V_{1}+v$ is equal to 16 and $V_{1}-v$ is equal to 12. According to Figure S1, 12 and 14 are in the third quartile and 16 is in the third quartile. In other words, the distance between 14 and 16 is more significant than the distance between 14 and 12, because the increase changes the quartile but the decrease does not. This important difference is not reflected in the value of a distance-based measure.

**Supplementary Definition 1**

Based on Practice 3 in the main text, let us consider a direct use of the definition of inversion for only one person. For person 1, sequence $S= \left\langle10, 6, 5, 1, 8 \right\rangle$. Here, the first element $a_{1}= 10$ (where $i$ is 1) is inverted with respect to the second element $a_{2}= 6$ (where $j$ is 2) because it occurs before it in the sequence ($i<j$) but its value is higher ($a_{i}>a_{j}$). Similarly, the same element is inverted with respect to the remaining three elements. Also, the second element $a_{2}= 6$ is inverted with respect to 5 and 1 and the third element $a_{3}= 5$ is inverted with respect to 1. Thus, in this sequence, there are seven inversions.

**Supplementary Code 1**

The algorithm for calculating the number of inversions is given in Code 1.

struct InvFeature

{

string Name;

int Value;

};

1 int InvCount(InvFeature[] array1, InvFeature[] array2)

2 {

3 sortByValue(array1);

4 sortByValue(array2);

5 int inv_count = 0;

6 for (int i = 0 to Length(array1) - 1)

7 if(GetFeatureIndex(array1,array1[i].Name) < GetFeatureIndex(array2,array1[i].Name))

8               inv_count++;

9   return inv_count;

10 }

Code S1. Algorithm for calculating the number of inversions

To calculate the number of inversions, in addition to the value of each feature, the name is also required. For this purpose, a new structure is defined as InvFeature. The algorithm takes two arrays as the input parameters and returns the number of inversions between the two arrays as the result. First, the input arrays are sorted based on the values using the sortByValue function (line2, line3). The sortByValue function takes an array as its only parameter and sorts this array based on the values. The number of inversions is calculated as the sum of the differences in the positions of each feature between the two sorted arrays. For this purpose, GetFeatureIndex is used. This function takes an array and the name of the feature as input parameters and returns the index of that feature in array as output parameter. In order not to calculate the number of inversions twice, the algorithm checks that the position of the feature in the second vector is greater than the position of the same feature in the first vector (line7).

**Supplementary Code 2**

The pseudo-code of the inversion-based clustering algorithm is given in Code 2.

# Inversion-Based Clustering Algorithm Clustering Pseudocode

def inversion-based_clustering(data, k, max_iterations):

# Step 1: Initialize cluster centroids randomly

centroids = initialize_centroids(data, k)

for iteration in range(max_iterations):

# Step 2: Assign each data point to the nearest centroid

clusters = assign_to_clusters(data, centroids)

# Step 3: Update centroids based on the number of inversions

centroids = update_centroids(data, clusters)

return clusters, centroids

# Function to initialize centroids randomly

def initialize_centroids(data, k):

centroids = randomly_select_k_points(data, k)

return centroids

# Function to assign each data point to the nearest centroid

def assign_to_clusters(data, centroids):

clusters = []

for point in data:

# Calculate number of inversions for each centroid

distances = [Inv(point, centroid) for centroid in centroids]

# Assign point to the cluster with the minimum number of inversions

cluster_assignment = argmin(distances)

clusters.append(cluster_assignment)

return clusters

# Function to update centroids based on the new clusters

def update_centroids(data, clusters):

new_centroids = []

for cluster_id in range(max(clusters) + 1):

# Extract points assigned to the current cluster

cluster_points = [data[i] for i in range(len(data)) if clusters[i] == cluster_id]

# Calculate the mean of the points in the cluster to get the new centroid

new_centroid = calculate_mean(cluster_points)

new_centroids.append(new_centroid)

return new_centroids

# Additional utility functions:

# - Inv(point1, point2): Calculate number of inversions between two points

# - randomly_select_k_points(data, k): Randomly select k points from the dataset

# - calculate_mean(points): Calculate the mean of a list of points

# - argmin(values): Return the index of the minimum value in a list

Code S2. The pseudo-code of inversion-based clustering algorithm

**Supplementary Practice 1**

One disadvantage of most similarity measures is that adding a value to a feature has the same effect on distance as subtracting the same value from it (Symmetry challenge). But in the inversion-based similarity measure, the effect on distance of adding a value to a feature may be different from the effect of decreasing by that value. This difference illustrated by Practice 1.

**Practice 1 (****Symmetry challenge)**: Suppose in Practice 3 in the main text that person 1 and movie 4 are selected as the reference object and the reference feature, respectively. First, 4 units are added to the reference feature of person 2 and the number of inversions is calculated. Alternatively, 4 units are subtracted and the number of inversions is recalculated. The other features do not change. The results are shown in the Table S1, Figure S2, Table S2, and Figure S3.

Table S1. Users' ratings of movies after adding 4 units to the reference feature of person 2

| Movies | Score of person 1 | Score of person 2 |
| --- | --- | --- |
| Movie1 | 10 | 9 |
| Movie2 | 6 | 3 |
| Movie3 | 5 | 9 |
| Movie4 | 1 | 6 + 4 =10 |
| Movie5 | 8 | 1 |


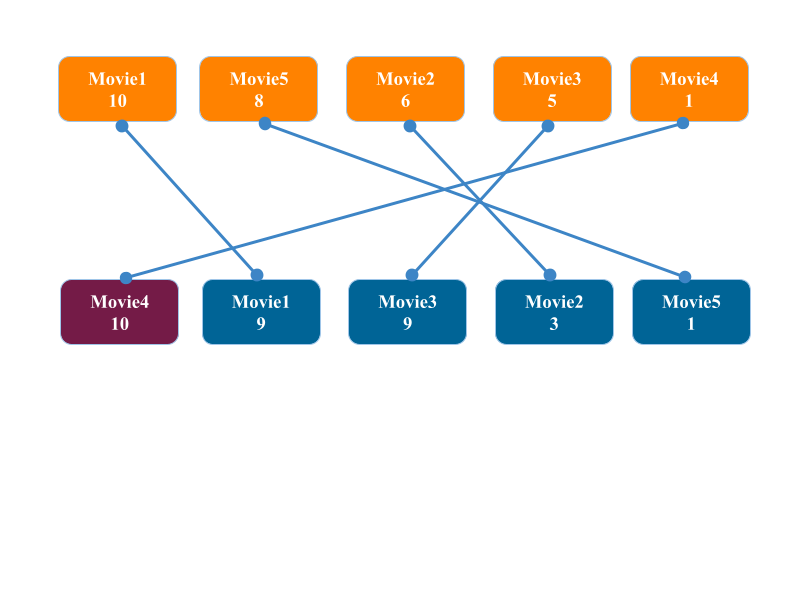


Figure S2. Number of inversions after adding 4 units to the reference feature of person 2

Table S2. Users' ratings of movies after subtracting 4 units from the reference feature of person 2

| Movies | Score of person 1 | Score of person 2 |
| --- | --- | --- |
| Movie1 | 10 | 9 |
| Movie2 | 6 | 3 |
| Movie3 | 5 | 9 |
| Movie4 | 1 | 6 - 4 =2 |
| Movie5 | 8 | 1 |


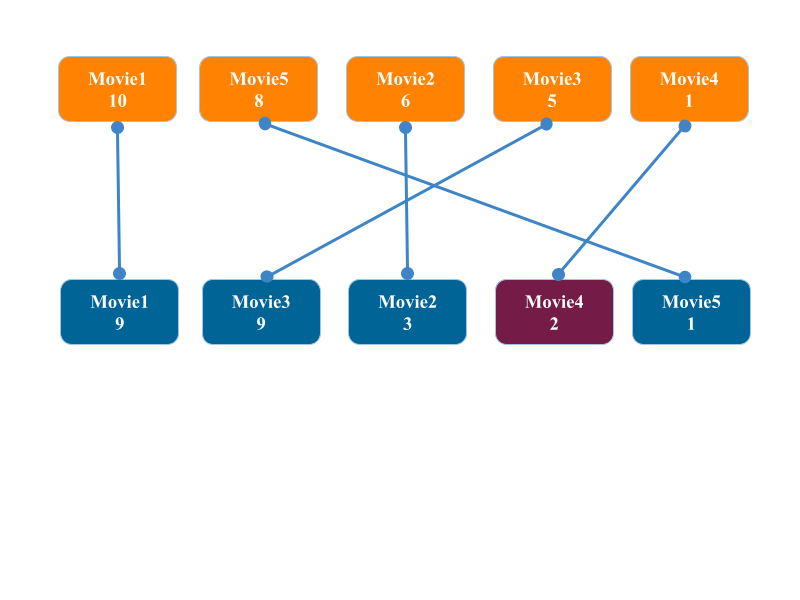


Figure S3. Number of inversions after adding 4 units to the reference feature of person 2

As shown in Figure S2 and Figure S3, adding or subtracting a value to an attribute can have a different effect on distance. Recall that the number of inversions (the number of points where the lines intersect) in Practice 3 in main text is 5. If 4 units are added to reference feature, the number of inversions becomes 7 but if 4 units are subtracted, the number of inversions becomes 4.

Another disadvantage of most similarity measures is that adding a value to one feature has the same effect on distance as adding the same value to another feature (Place Symmetry challenge). The proposed measure also addresses this challenge. In other words, the effect on number of inversions of adding a value to a feature may be different from the effect of adding that value to another feature.

**Supplementary Documentation 1**

Synthetic datasets were generated using the MixSim package, which is designed for generating Gaussian distribution mixtures with varying degrees of overlap between their components. Clustering complexity in these datasets hinges on the overlap parameter, dictating interaction levels among mixture components. The overlap parameter is determined by pairwise overlap, computed as the sum of two misclassification probabilities. According to the package documentation, the average pairwise overlap measure (denoted as ῶ) typically ranges from 0.4, indicating extreme overlap between clusters, to 0.001, indicating very low overlap. For further exploration of the impact of the ῶ parameter on data generation and its influence on creating clusters with varying degrees of complexity, ranging from well-separated to highly overlapped ones, see **Supplementary Simulation S1** [see Additional file 1].

The previous section introduced two algorithms (ICA and RIECA) using the inversion-based similarity measure for clustering. Here, we present simulation experiments assessing the effectiveness of these algorithms. Results of these simulations are detailed in **Supplementary Simulation S2** and **Supplementary Simulation S3** [see Additional files 1].

Two main categories of evaluation measures exist for assessing clustering algorithms: unsupervised and supervised. Unsupervised measures evaluate cluster quality solely based on the data, without additional information. In contrast, supervised measures use additional data to evaluate clustering results. This additional data specifies the correct cluster assignment for each object being clustered. By comparing this information with the clusters produced by the algorithm, we can assess how accurately the data is placed in the correct clusters.

A prominent supervised evaluation measure is the *Rand index*, calculated as the ratio of correctly classified pairs of objects to the total number of pairs. The Rand index's values range from 0 to 1, with higher values indicating better cluster quality. Another measure, the *adjusted Rand index*, considers chance grouping of objects, akin to the *Rand index*.

**Supplementary Simulations**

**Simulation S1 (the effect of values of ῶ on generating overlapped and separate clusters):**

The results of generating 30 random data values in three clusters with different values of ῶ are shown in Figure S4. The clusters are shown in three colors: blue, green, and red. The figure shows the effect of values of ῶ on the nature of the clusters that are generated. For high values of ῶ, the clusters are highly overlapped, while for low the values, the clusters are well separated.


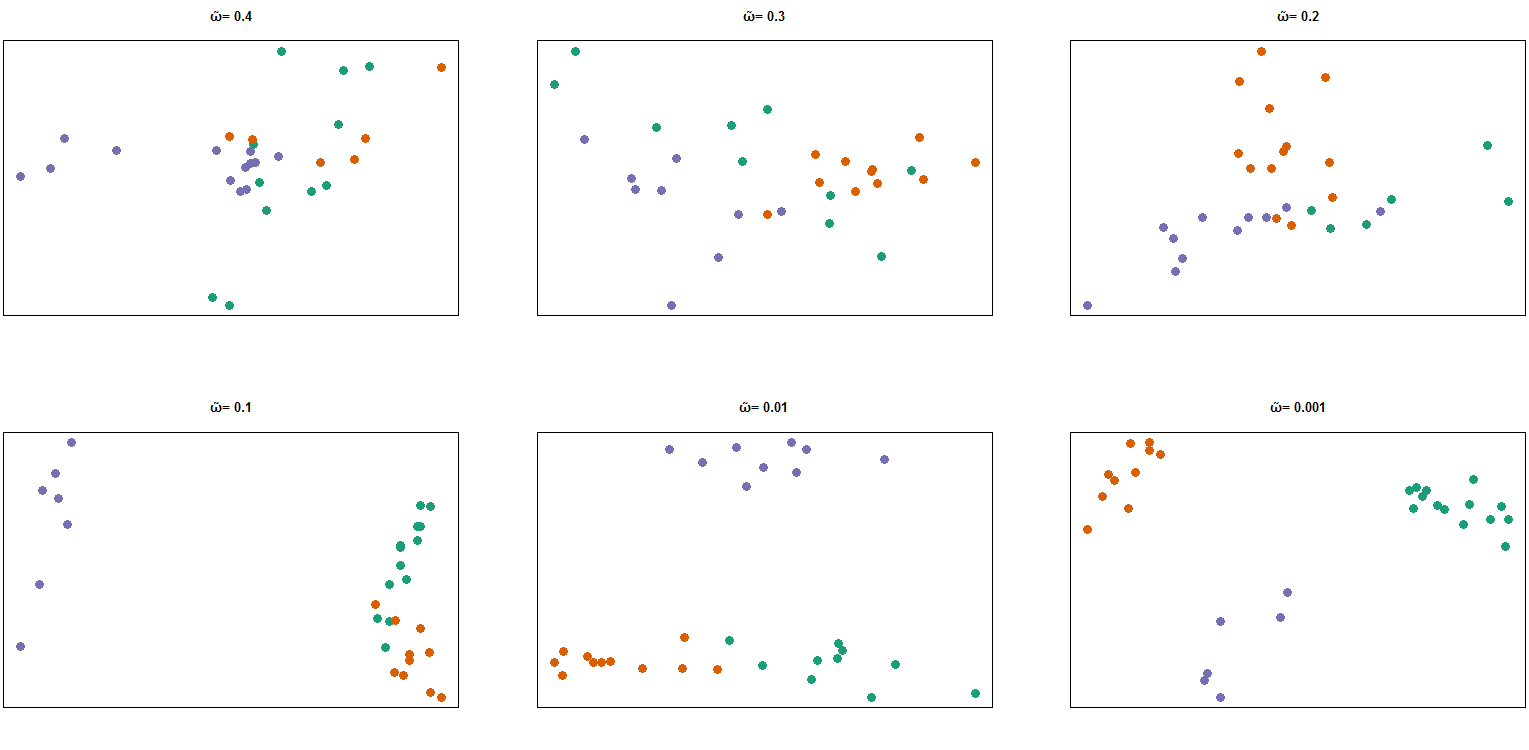


Figure S4 : The effect of parameter ῶ when generating clusters

**Simulation S2 and S3**

Two simulation experiments were performed to provide evidence for the claim that the proposed measure addresses the three mentioned challenges in the study.

For this purpose, 30 objects $\left\langle{obj}_{1},{obj}_{2},\ldots,{obj}_{30} \right\rangle$with 8 features $\left\langle f_{1},f_{2},\ldots,f_{8} \right\rangle$ were created randomly. ${obj}_{1}$ was selected as the reference object.

**Simulation S2 (Symmetry challenge)**: To perform this experiment, $f_{2}$ was selected as the reference feature. The number of inversions of the reference object with other objects was first calculated. Then, for all objects, a value $v$ was added to$f_{2}$ and the number of inversions was recalculated. It was assumed that $S_{i}$ is the same for all features $1<i<8$, and is equal to [0, 1]. When adding $v$ to $f_{i2}$ causes it to be greater than 1, the result is modified to $1-(f_{i2}+v)$. So, without loss of generality we assume that:

$$f_{12} ,f_{i2}+v \in S_{f2} (S1)$$

*where*

$$2\leq i\leq30$$

This simulation was performed 500 times and the mean and standard deviation were calculated. The average change in the number of inversions (denoted$delta \Delta$) is shown in Table S3.

Table S3 : The average change in the number of inversions (Symmetry challenge)

| Value ($\boldsymbol{v}$) | 0.05 | 0.1 | 0.3 | 0.6 |
| --- | --- | --- | --- | --- |
| $\boldsymbol{delta}$ | $0.86\pm0.67$ | $1.33\pm0.68$ | $1.89\pm0.48$ | $1.87\pm0.46$ |

**Simulation S3 (Place Symmetry challenge):** The only difference between this experiment and the previous one is that in each simulation run the addition of value $v$ is applied to a randomly chosen feature. The average change in the number of inversions is shown in Table S4.

Table S4: The average change in the number of inversions (Place Symmetry challenge)

| Value ($\boldsymbol{v}$) | 0.05 | 0.1 | 0.3 | 0.6 |
| --- | --- | --- | --- | --- |
| $\boldsymbol{delta}$ | $0.95\pm0.88$ | $1.41\pm0.79$ | $2.59\pm0.64$ | $2.78\pm0.57$ |

As shown in Table S3 and Table S4, any change of 0.05 or more in feature values causes a change in the number of inversions. As shown in Figure S5, there is a positive relationship between the added value $v$ and the average change in the number of inversions.


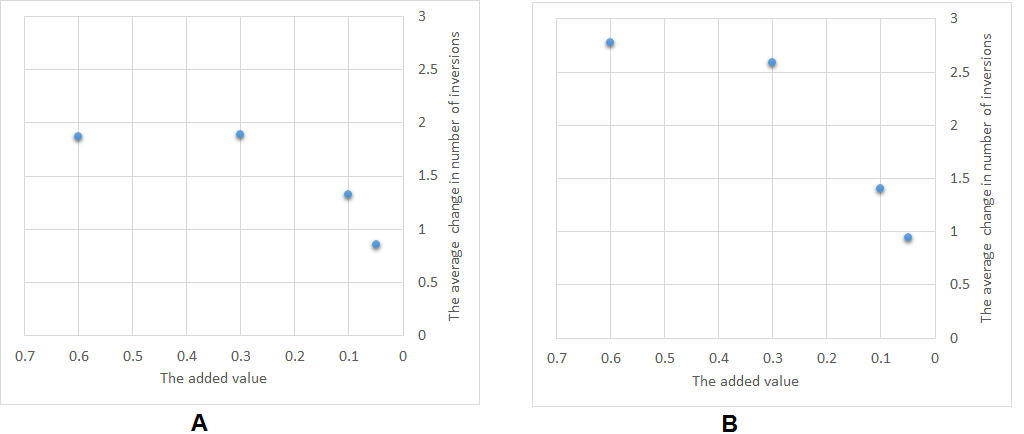


Figure S5: Scatterplot of the added value v in relation to the average change in the number of inversions. A) Symmetry challenge B) Place Symmetry challenge

**Supplementary statistical test result 1**

Table 4 in main manuscript only shows that the average values of the adjusted Rand index for the algorithms are different. In order to evaluate the statistical significance of the observed differences, a one-way ANOVA statistical test was used. The results of the statistical test for ῶ = 0.4 are stated below.

The null and alternative hypothesizes are as follows:

$$H_{0}: \mu_{AR(k-means)}= \mu_{AR(EM)}= \mu_{AR(Hierarchal)}=\mu_{AR(k-medoids )}=\mu_{AR(ICA)}=\mu_{AR(REICA)}$$

$$H_{1}: The mean of at least one algorithm is different from the others (S2)$$

The results of the statistical test are shown in Figure S6.


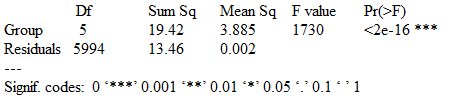


Figure S6: One-way ANOVA result (ῶ = 0.4)

There was a significant difference in the mean adjusted Rand index [*F* (5, 5994) = 766.8, *p* < 0.001] between the algorithms. *Post hoc* tests with the Tukey test were carried out to confirm where those differences are. The results are shown in Figure S7.


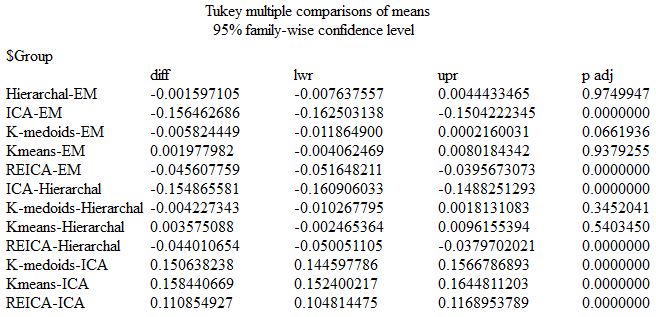


Figure S7: Result of post hoc tests (ῶ = 0.4)

Due to the large number of comparisons made, only the results related to REICA are expressed. There was a significant difference between REICA and k-means (p < 0.001) with the adjusted Rand index for k-means an average of 0.044 more than for REICA. There was also a significant difference between REICA and EM (p < 0.001) with the adjusted Rand index on EM an average of 0.055 more than for REICA. There are similar results for k-Mediods and Hierarchical with differences of 0.049 and 0.052, respectively. One important point in the above comparisons is that there is no significant difference between ICA and REICA. Another point is that the difference between EM, k-mediods, and Hierarchical is not significant. According to the statistical tests, the ranking of algorithms based on effectiveness is shown in Table S5.

Table S5: Ranking of algorithms based on effectiveness (ῶ = 0.4)

| Algorithm | Rank (ῶ = 0.4) |
| --- | --- |
| EM clustering | 3 |
| k-means clustering | 2 |
| k-medoids clustering | 3 |
| Hierarchal clustering | 3 |
| ICA | 1 |
| REICA | 1 |

Table S6 shows the most effective algorithm for each value of ῶ.

Table S6: The most effective algorithm in clustering 6-dimensional datasets of size 200 with 5 clusters

| ῶ | The most effective algorithm |
| --- | --- |
| ῶ = 0.4 | REICA, ICA |
| ῶ = 0.3 | REICA |
| ῶ = 0.2 | REICA |
| ῶ = 0.05 | k-means |
| ῶ = 0.001 | k-means, k-means, Hierarchical |

A similar process was performed for the second experiment. The results are shown in Table S6 and Table S7.

Table S7: Effectiveness of the algorithms in clustering 7-dimensional datasets of size 500 with 10 clusters. The most effective algorithm is highlighted in gray.

|  | 0.4 | 0.3 | 0.2 | 0.05 | 0.001 |
| --- | --- | --- | --- | --- | --- |
| EM clustering | $0.034\pm0.010$ | $0.058\pm0.016$ | $0.108\pm0.025$ | $0.429\pm0.059$ | $0.974\pm0.019$ |
| k-means clustering | $0.041\pm$ 0.011 | $0.073\pm0.016$ | $0.134\pm0.026$ | $0.474\pm0.051$ | $0.945\pm0.053$ |
| k-medoids clustering | $0.038\pm$ 0.010 | $0.064\pm0.014$ | $0.115\pm0.023$ | $0.406\pm0.052$ | $0.959\pm0.010$ |
| Hierarchal clustering | $0.035\pm$0.009 | $0.061\pm0.14$ | $0.112\pm0.023$ | $0.415\pm0.052$ | $0.966\pm0.018$ |
| ICA | $0.055\pm0.010$ | $0.085\pm0.015$ | $0.133\pm0.022$ | $0.343\pm0.046$ | $0.770\pm0.077$ |
| REICA | $0.058\pm0.011$ | $0.091\pm0.016$ | $0.148\pm0.025$ | $0.424\pm0.050$ | $0.924\pm0.033$ |

Table S8: The most effective algorithm in clustering 7-dimensional datasets of size 500 with 10 clusters

| ῶ | The most effective algorithm |
| --- | --- |
| ῶ = 0.4 | REICA |
| ῶ = 0.3 | REICA |
| ῶ = 0.2 | REICA |
| ῶ = 0.05 | k-means |
| ῶ = 0.001 | $\mathrm{EM}$ |
